# COVID-19 Pandemic Control Measures and Their Impact on University Students and Family Members in a Central Region of Spain

**DOI:** 10.3390/ijerph20054470

**Published:** 2023-03-02

**Authors:** Lucía Pérez-Pérez, Inés Cárdaba-García, Miguel A. Madrigal-Fernández, Federico Montero-Cuadrado, E. M. Sobas, Raúl Soto-Cámara

**Affiliations:** 1 Nursing Department, Faculty of Nursing, University of Valladolid, 47005 Valladolid, Spain; 2 Health Service of Castilla y León (Sacyl), 47007 Valladolid, Spain; 3 East-Valladolid Primary Care Management (Sacyl), 47010 Valladolid, Spain; 4 Unit for Active Coping Strategies for Pain in Primary Care, 47011 Valladolid, Spain; 5 Institute of Applied Ophthalmobiology (IOBA), University of Valladolid, 47011 Valladolid, Spain; 6 Department of Health Sciences, University of Burgos, 09001 Burgos, Spain; 7 Emergency Medical Service of Castilla y León (Sacyl), 47007 Valladolid, Spain

**Keywords:** COVID, SARS-CoV-2, pandemics, universities, prevention, suicide

## Abstract

The first waves of the COVID-19 pandemic were times of great change in the lives of university students and their families in Spain. The aim of this study was to explore the psychosocial aspects and preventive measures carried out during the COVID-19 pandemic by students and family members of the nursing degree students of the University of Valladolid (Spain). The number of people surveyed was 877, by means of an ad hoc questionnaire. Relationships between variables were established by means of the Chi-square test and Student’s *t*-test. In addition, multivariate logistic regression was generated. The significance level used was 0.05. Students and family members maintained preventive measures= such as hand washing, correct use of masks =in closed places, avoiding crowds and maintaining social distance, but at low rates (close to 20% in all cases). Regarding psychosocial aspects, 41.07% of the participants suffered from anxiety and loneliness, while 5.2% needed to take drugs to reduce anxiety or sleep and 66.07% were dependent on technology. Suicidal behavior is related to stress, anxiety, loneliness, poor family relationships, psychotropic drug use and technology abuse. The pandemic has caused life changes in university students and their families at the psychosocial level, generating high figures of suicidal ideation regardless of age. Preventive measures adopted to control the pandemic have not been followed for the most part.

## 1. Introduction

The COVID-19 pandemic has brought about many profound changes in the daily habits of millions of people. Many of these changes are due to the containment conditions implemented in all countries of the world [1]. In Spain, a state of alarm was declared on 14 March 2020, and restrictive measures on fundamental activities, such as people going out on the streets, were adopted, alongside the suspension of all leisure, cultural and sporting activities. University students in the province of Valladolid were unable to attend university in person, switching to online teaching [2]. Therefore, university students changed their practices very abruptly, and without a period of adaptation, to a new model of virtual teaching [3] during the first and second waves of COVID 19 when the study was carried out. On 21 June, after 98 days, the state of alarm ended and Spain entered the so-called “new normal”. In the summer of 2020, there were multiple outbreaks in different areas of the country, which degenerated into community transmission. On 21 October 2020, Spain exceeded one million infections. On 25 October 2020, the Spanish government decreed a second state of alert to deal with the second wave of infections [4].

Coronavirus disease or COVID-19 was the cause of a high number of deaths in the first months of its appearance, wherein policy makers and health authorities were forced to take urgent measures to try to curb the pandemic [5]. These measures have influenced both university students and their families.

According to data from the Spanish Ministry of Health, as of 31 December 2022, the COVID-19 pandemic has caused 117,095 deaths [6]. According to official figures, the number of people who have been confirmed cases of COVID-19 in Spain is 13,684,258. As of mid-June 2020, Spain was the fifth country in numbers of confirmed cases, behind the United States, Brazil, Russia, and the United Kingdom, and the sixth country in number of deaths, behind the United States, Brazil, the United Kingdom, Italy and France [7].

A feature of COVID-19 that differs from other respiratory infections is its multisystem symptomatology, complications, and long-term sequelae [4]. The most prevalent COVID-19 symptoms and sequelae in patients overall were fatigue, dyspnea, cough, anosmia, agues, and joint pain. Many of the symptoms were persistent at 30, 60 and more than 90 days after symptom onset [8].

Diagnostic testing for COVID-19 disease had a major impact on healthcare institutions. Rapid diagnosis was important for early isolation, contact tracing, preventive measures, and infection control [9,10]. Although polymerase chain reaction (PCR) was and still is the general standard, other means of diagnosis had to be sought, as it is very costly, time-consuming and requires special equipment and skilled technicians. Rapid diagnosis through antigen detection (Ag-RDT) is useful and can detect the presence of the SARS-CoV2 virus in respiratory samples within 20 min, and even without the need for a laboratory [11].

Several vaccines are now available and are essential to reduce the spread of the disease and protect public health. Countries around the world have implemented several social distancing policies, an important factor in reducing human-to-human contact and thus transmission of the virus [12]. The Spanish Ministry of Health established the 6 M strategy to facilitate the recall of the measures necessary to avoid SARS-CoV-2 transmission, which are masks covering the mouth and nose, distance meters (1.5 m), frequent hand washing, less contact when moving in stable bubble groups, more ventilation (with outdoor activities and open windows when indoors) and staying at home if you have symptoms, have been diagnosed with COVID-19, are a contact, or are awaiting results [13].

The COVID-19 pandemic has brought a risk of psychological illness and sleep disorders to the entire population [14,15], including young university students, because people have been consistently exposed to bad news, which generates stress and anxiety [16,17]. The loss of millions of lives due to COVID-19 placed many families in a state of bereavement. In a study conducted at the San Cecilio Hospital in Granada (Spain), anxiety and depression manifested up to two months after the loss of a family member or friend. This study found an association between bereavement and levels of anxiety and depression [18,19,20].

Another study showed that the loss of a family member produces a series of neuropsychological changes, such as alterations in the neurocognitive system and the neural system involved in emotional regulation, thus demonstrating the association between loss and psychological distress [18].

Homes during the pandemic have in many cases become places of work, study, recreation and leisure. This has led to the need for changes in health self-care behaviors in the face of COVID-19 [19,20] through undertaking activities to adapt to new lifestyles [21,22].

Mental illnesses such as anxiety and stress are problems that can be experienced by anyone regardless of race, gender or age. According to the WHO’s latest Global Burden of Disease study, the pandemic has affected the mental health of young people, who are disproportionately at risk of suicidal and self-harming behavior. It also indicates that women have been more affected than men and were more likely to develop symptoms related to mental problems [23].

Anxiety and stress alter the quality of life of those who suffer from them [24]. Studies show that university students were the main group affected by these mental illnesses during the COVID-19 pandemic [25,26,27]. Studies by Forte, Favieri, Tambelli and Casagrande concluded that individuals suffered from psychopathological symptoms, anxiety and post-traumatic stress symptoms after the first waves of COVID-19 [28]. In addition, suicidal ideation has increased considerably with the COVID-19 pandemic. This factoris part of the cognition of the individual, who can communicate his or her intentions and plans to carry out the suicidal act [29,30].

Therefore, the following study objective was proposed: to determine the preventive measures taken against COVID-19 infection, the COVID-19 screening and diagnostic tests used and their impact on the mental health of students at the University of Valladolid and their families, focusing on the presence of suicidal ideation, stress, anxiety, loneliness, family disputes, drug use and technology dependence, and taking into account age.

## 2. Materials and Methods

### 2.1. Study Design and Sample

A descriptive cross-sectional study was conducted in 2nd year students of the nursing degree at the University of Valladolid (Spain) and their first and second degree relatives. Second-year nursing students were included in this study because they were those who were not yet carrying out clinical practice in hospitals and had started their university studies at the beginning of the COVID-19 pandemic, so a priori they could be considered the most affected. The study was conducted between 1 October 2021 and 31 December 2021.

The total population included in this study was 877 people, with a response rate of 87%.

The inclusion criteria for participation were:

(a) Students enrolled in the 2nd year of the Bachelor’s degree in nursing at the Faculty of Nursing at the University of Valladolid in the academic year 2021–2022.
(b) Voluntarily participation without financial remuneration.
(c) Relatives of first and second degree of consanguinity of students enrolled in the second year of the degree in nursing at the University of Valladolid during the academic year 2021–2022, and living together during the first confinement of the SARS-CoV-2 pandemic.
(d) Responses to all the questions in the survey, and authorized informed consent to participate in the study, in accordance with the Declaration of Helsinki.

### 2.2. Procedure

The data were obtained by means of a survey via a digital link on the Google Forms^®^ platform that was distributed online through the virtual campus of the University of Valladolid, and the enrolled students passed it on to their first and second degree relatives living with them during the first confinement of the SARS-CoV-2 pandemic.

At the beginning of the questionnaire, potential participants were informed of the object of the study, the implication of their participation as well as the possibility of resolving doubts, through an e-mail address. They were then asked for their free, voluntary and informed consent to respond to the survey, as well as a responsible declaration of compliance with the inclusion criteria.

In any case, the process was anonymous, allowing withdrawal from the study at any time. The research protocol was approved by the Ethics and Drug Research Committee of the Valladolid East Health Area (PI 22-2542), respecting the principles of the Declaration of Helsinki.

### 2.3. Study Variables

An “ad hoc” questionnaire was used as a data collection instrument. In order to check that the survey was valid for the study, it was evaluated by a committee of expert researchers from the Department of Nursing of the University of Valladolid, who made the necessary modifications for a better understanding of the questions. The estimated completion time was approximately 10 min.

This questionnaire inquired about socio-demographic variables (age, sex, type of housing, residential environment, nationality and level of education), screening tests, diagnosis, quarantine status and preventive measures taken during hospitalization due to the COVID-19 pandemic. The psychosocial aspects experienced by the students and their families were also analyzed in terms of anxiety and depression as well as suicidal ideation. For this assessment, participants are asked to state whether they have an anxiety disorder or depression only if they have a diagnosis issued by a health professional.

### 2.4. Statistical Analysis

For the between-group comparison, participants were classified into three age groups in the same way as Fantin et al. [31]. Finally, they were redistributed into two groups due to a lack of sample size in one of them. Group 1 consisted of people aged 18–39 years and Group 2 of people aged 40–68 years.

Qualitative variables were described by their frequencies and percentages, while quantitative variables were described by mean and standard deviation (SD). Normality criteria for quantitative variables were assessed using the Kolmogorov–Smirnov test. Comparison between variables was performed by the Chi-squared test and Student’s *t*-test for independent samples, depending on the nature of the variables. For multiple comparisons, multivariate logistic regression analysis was performed. Variables that reach statistical significance in the bivariate analysis are included in the model. Statistical significance was considered if *p* ≤ 0.05. Statistical analysis was performed with SPSS version 26.0 software (IBM-Inc, Chicago, IL, USA).

## 3. Results

**Figure 1:**
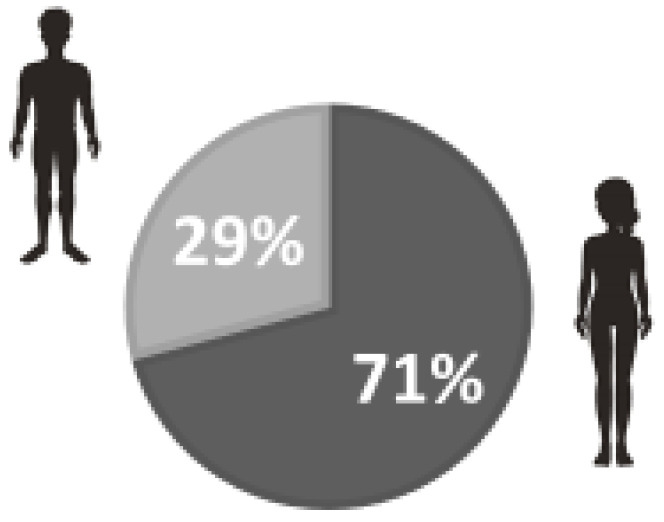
Distribution of the sample by gender (men and women).

**Figure 2:**
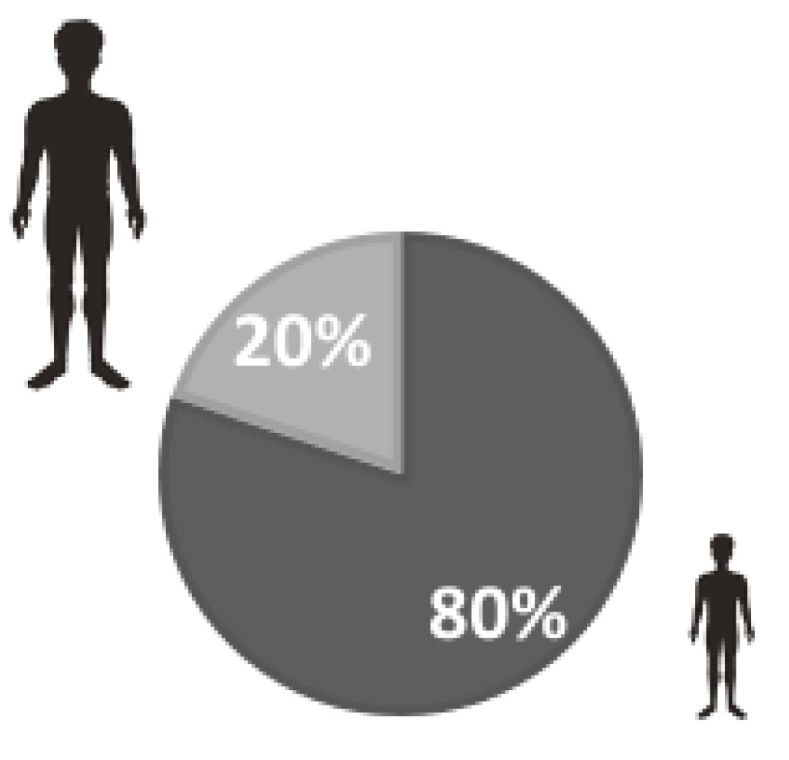
Distribution of the sample by age (Group 1: ≤39 years; Group 2: ≥40 years).

The study sample consisted of 877 people whose mean age was 27.25 years (±13.79). By age group, the sample included 703 people under 39 years of age (80.5%), 142 between 40 and 59 years (16.3%) and 32 over 60 years (3.2%). Of the total, 617 were women (70.4%) (Figure 1), 556 lived in a flat larger than 50 m (63.4%), and 594 (67.7%) lived in a rural environment. The mean number of cohabitants was 3.49 (±1.02). 98.6% were of Spanish nationality. The most frequent level of education was university studies (57.4%), followed by intermediate studies (36.7%). The distribution of the sample characteristics in terms of screening tests, diagnosis and quarantine status related to COVID-19 by age is summarized in Table 1, according to two age groups (Group 1: under 39 years; Group 2: over 40 years). Figure 1 represents the distribution of the sample by gender, the sample being mostly female. Figure 2 shows the sample based on age, where those under 39 are the most numerous.

The measures that were taken in relation to precautions to prevent infection with SARS-CoV-2 were analyzed on the basis of the marked age groups and the variable male and female gender, as there were no responses in the sample that indicated that anyone identified with another gender. This distribution can be found in Table 2.

Aspects related to vaccination were studied on the basis of the age of the participants. According to this criterion, age was related to being vaccinated against COVID-19 (*p* = 0.011), to being vaccinated against influenza (*p* = 0.001), and to belonging to a group at risk of COVID-19 infection (*p* < 0.000).

With regard to mental health during the first period of confinement in the study sample, we inquired about different feelings and situations that might be present in the participants who reported suicidal ideation. Table 3 shows the relationship of these variables with the age group to which the respondents belong.

The situation of the health system in Spain was rated differently by the participants depending on whether they were men or women. This is the case with the opinion on whether other chronic diseases were being adequately treated or were being neglected due to the high demand for COVID-19 patients (*p* = 0.010), and the feeling of having had a family member who had not been adequately cared for due to being immersed in the COVID-19 pandemic (*p* = 0.017). There were no differences in opinion by gender with regard to the assessment of the Spanish health system in comparison with that of other countries (there was a negative response from 52.3% of the sample) and whether or not real official figures were being given for the number of infections and deaths (there was a negative response from 79.8% of the sample).

Finally, the presence of persistent symptoms of COVID-19 was studied. Of the participants who suffered from the disease, 20.2% were diagnosed less than one month ago, 16.0% between one and two months ago, 19.1% between two and three months ago, 13.8% between three and five months ago and 30.9% more than five months ago at the time of completing the survey. Sequelae after overcoming the infection were present in 26.2% of men and 53.7% of women. Of the list of sequelae, in both men and women, the most frequent were those related to fatigue or tiredness derived from the infection (2.0% vs. 3.4%).

The distribution of sequelae according to age showed no statistical differences in the case of digestive (*p* = 0.788), respiratory (*p* = 0.606), neurological (*p* = 0.737), cardiac (*p* = 0.959), fatigue (*p* = 0.400) or psychological effects (*p* = 0.973).

## 4. Discussion

This study evaluated compliance with COVID-19 pandemic control measures according to age group, as well as the impact of the pandemic on the mental health of second-year undergraduates of the nursing degree in Valladolid (Spain) and their families.

The average age of the sample is young (27.25 years), which makes it possible to compare the results with those obtained by other authors in this type of population. This is the case for university students in Bangladesh, whose average age is similar to that of this study [32].

The sample is mainly female, due to the fact that the Nursing Degree is feminized. Even so, there is a representation in line with the volume of male students who are usually enrolled in these studies [33].

The average number of cohabitants is between three and four, which suggests that in many cases, nursing students lived with their family members during their confinement. A survey carried out in the Chinese population indicates that if family cohabitation is positive and supportive, it promotes individual and family well-being, so people in the sample, if they have had such conditions, have been protected; those who have no communication with family are the ones who have most often needed psychological help [34].

There are hardly any percentage differences in SARS-CoV-2 infection in the two age groups in the sample, which allows us to dispel the idea that young people have been infected more than older people in the early stages of the pandemic. Another Chinese study shows how false reports have spread through the media, in some cases leading to stigmatization of groups such as the younger population, without any demonstrable reality [35].

In the sample of this study, the most commonly used diagnostic test was PCR, followed by antigen determination, as in other cases [36,37]. Considering that PCR is highly sensitive and specific for detecting viral RNA compared to other methods, it is logical that it is used more frequently [38]. It is true that in the early stages of the pandemic, less reliable methods such as antigen detection, which is much faster and does not require specialized personnel, were used more frequently, but as virus transmission has been controlled, PCR has become the diagnostic technique par excellence [39].

Home confinement has posed difficulties in some cases, as it has not always been easy to avoid contact with family members due to the impossibility of strict isolation. The same result has been obtained in the UK population [40]. Moreover, it is striking that in many cases, there has been a fear of being discriminated against for having to be isolated. This situation has been extensively studied in nurses and other health professionals who worked during the pandemic, but not in nursing students, who stopped their clinical practice in the early stages of the pandemic, due to the insecurity experienced [41].

Younger people report that the place where they remove their masks with friends is on the terrace of a bar, followed by at home. Older people, on the other hand, tend to remove their masks mainly at home, followed by in a restaurant. A population study in Australia confirms that errors in mask use during the COVID-19 pandemic are frequent, and that more education on mask use is needed for the population [42].

Both young and older people almost entirely report taking measures before and after leaving home, indicating a fear of bringing infection home. Ruhnke studied the impact of prolonged use of these measures and concluded that it leads to so-called precaution fatigue which negatively affects the health of the population [43].

It is also noteworthy that younger people frequently have unmasked contact with 3–4 people, while older people have unmasked contact with 5–6 people. It is not known whether these contacts with more people are due to their work environment. Further studies are needed to clarify this aspect.

In relation to the type of mask used, younger patients tend to use surgical masks, while older patients use surgical masks and FFP2 almost equally. In Germany, the risks and benefits of each type of mask were studied, suggesting that those who are older or susceptible to complications in case of contact should frequently use FFP2. However, this research is limited as it is not known whether at-risk personnel used one or the other type of mask in this research [44].

The use of ozone is a measure that has hardly been used by the population, while the use of masks, avoiding crowds, limiting kissing and hugging, frequent hand washing and limiting contacts are all carried out in similar numbers by around 20% of the sample, which is quite a low proportion. Considering the results of Joob and Wiwanitkit, which indicate that there is no relationship between the level of ozone in a facility and the level of SARS-CoV-2, it is logical that use has been minimal; this is also because of the higher cost of ozone compared to other measures [45] against COVID-19. Chinese researchers found that the virus has a high transmissibility and is rapidly transmitted between people through close contact and droplets from coughing, sneezing and loud talking as well as through contact with contaminated objects; therefore, it makes sense to limit greetings and contacts [46]. Hand washing has been shown to be an effective personal protection technique to prevent transmission of SARS-CoV-2 in unvaccinated persons, but its effect is more limited in vaccinated persons [47].

Age influences eligibility for vaccination, so those who are vaccinated for influenza and are at risk of severe disease if they become infected with SARS-CoV-2 are more likely to be vaccinated; therefore, it is likely that older people are more likely to have been vaccinated for COVID-19. Silva et al. found that the young university population would be willing to be vaccinated for COVID-19 if the vaccine were shown to be safe and effective [48].

The increased incidence of suicides and suicide attempts during the pandemic is well known [49,50]. Farooq et al. find that the main risk factors for suicidal ideation were low social support, high physical and mental exhaustion and poorer self-reported physical health, sleep disturbances, quarantine and exhaustion, loneliness, and mental health problems. In the case of the sample, suicidal ideation is present in worrying numbers. The variables influencing the presence of suicidal ideation in this case are the same for both age groups. They are stress, anxiety, loneliness, arguments at home, use of psychotropic drugs and abuse of new technologies. Many of these are consistent with the aforementioned study [49]. This finding makes it possible to generate programs to care for the mental health of the sample and similar population groups in order to prevent completed suicides. There is a pattern of at-risk individuals that has nothing to do with age. Clearly, people with symptoms of stress and anxiety, who use psychotropic drugs or who have a behavioral dependence on new technologies are more at risk. People living in loneliness should be approached either by planning phone calls, through volunteering or through other actions, depending on available resources [51]. A poor home environment is also an important risk factor to consider [51]. Families have spent much more time together than usual, and this has led to frequent arguments [52]. Health promotion units should insist on programs that promote good treatment at home and positive communication.

Women were more likely to perceive that care for the chronically ill was neglected and that family members were more likely to be left to die. This may be due to the gendered role of caregiving that society often imposes on women [53], but further research is needed to provide more data on this. Women are also the most frequent sufferers of long-term symptoms, with fatigue being the most common [54]. Despite this finding, it is not infrequently stated that being male is a risk factor for death from COVID-19, but not as often it is stated that women are more likely to suffer from long-term symptoms. It is possible that there is a gender bias in health [55].

The population sample in general does not trust that officially reported figures are correct, indicating a lack of trust in the health system. The same result was obtained in a UK population study [56].

In Spain and China, previous studies have shown that in all psychological factors related to the COVID-19 pandemic, the individual differences of each person must be taken into account, and risk factors must not be overgeneralized [57,58].

This study has several limitations. The first limitation is that it was not possible to determine a causal relationship between variables due to the descriptive nature of the study. Another limitation is the lack of a comparison group with other undergraduate students and their relatives. It should be taken into account that in university health studies, the majority of students are female, and these results could be extrapolated to this type of population (female university students in health studies). However, they cannot be extrapolated to the general university population. The fact that this is a study based on a self-report administered online may lead to self-selection bias. Asking about an event that happened a year ago could cause recall bias in respondents. Finally, the scarcity of similar studies has made it difficult to compare and contrast the results obtained.

From a practical perspective, it would be advisable to create psychological support programs for the university population and their families, because the confinement prompted by COVID-19 can have academic, health and social repercussions. Even though the pandemic is on the decline, the risk of suicide in the population is still high, because it is associated with mental problems generated by the traumatic event. Moreover, these problems can affect students’ academic performance.

The article adds a novel aspect to the scientific literature, as health professionals have been extensively studied during the pandemic, but students studying a health degree at university have not, even less so in combination with their close relatives.

A future line of research would be to re-evaluate the same variables in other university students who do not belong to the health sciences, to determine if there are similarities and differences between them.

## 5. Conclusions

The confinement experienced during the first wave of the pandemic led to changes in the lives of university students and their families.

The most commonly used preventive measure was the use of surgical masks and hand washing, followed by changing clothes when entering the house, and finally, disinfecting footwear.

The screening and diagnostic tests carried out were PCR. A high percentage of students and family members had to maintain quarantine because they were diagnosed with COVID-19 or were close contacts.

Suicidal ideation presents as a mental health problem that should be addressed early in order to prevent it from being acted on. Psychological support is therefore essential for university students and their families to be able to adapt to a traumatic event. Early diagnosis and early intervention are the basis of adequate educational, personal, and professional performance in students.

## Figures and Tables

**Table 1:** Description of the characteristics of the sample in terms of screening tests, diagnosis and quarantine situation in relation to COVID-19 by age groups.

	Age Groups	
	Group 1 (≤39 Years) *n* (%)	Group 2 (≥40 Years) *n* (%)
**COVID-19 diagnosis** Yes No	64 (9.10) 639 (90.90)	19 (11.17) 151 (88.83)
**COVID-19 tests** PCR for COVID-19 COVID-19 antigen test COVID-19 antibody test COVID-19 serology	283 (54.32) 84 (16.12) 93 (17.85) 61 (11.71)	79 (43.17) 36 (19.67) 35 (19.12) 33 (18.04)
**Need quarantine** Yes No	450 (64.01) 253 (35.98)	84 (49.41) 86 (50.59)
**Quarantine difficulties** Not being able to isolate from family No isolation; mental problems Fear of discrimination No isolation; carer No isolation; late contact status	23 (38.33) 7 (11.66) 17 (28.33) 0 (0) 13 (21.66)	8 (47.06) 0 (0) 5 (29.41) 1 (5.88) 3 (17.65)

Abbreviations: PCR = polymerase chain reaction.

**Table 2:** Description of protection measures adopted against COVID-19 infection according to age and gender.

	Groups by Age and Gender			
	Group 1 (≤39 Years)		Group 2 (≥40 Years)	
	Male *n* (%)	Female *n* (%)	Male *n* (%)	Female *n* (%)
**Keep mask on with friends** Yes No	67 (35.26) 123 (64.74)	150 (29.41) 360 (70.59)	45 (70.31) 19 (29.69)	85 (81.73) 19 (18.27)
**Mask removal site (alone or combined)** Bar/Terrace Beach Swimming pool Disco or Bar Restaurant Address Labor sphere	115 (24.06) 61 (12.76) 52 (10.89) 40 (8.36) 87 (18.20) 111 (23.22) 12 (2.51)	327 (23.87) 208 (15.19) 174 (12.70) 99 (7.22) 245 (17.89) 310 (22.62) 7 (0.51)	9 (18.75) 6 (12.50) 5 (10.42) 0 (0) 11 (22.91) 13 (27.08) 4 (8.34)	9 (17.65) 9 (17.65) 5 (9.80) 0 (0) 14 (27.45) 12 (23.53) 2 (3.92)
**Measures when entering and leaving home** Yes No	167 (87.89) 23 (12,11)	485 (95.10) 25 (4.90)	62 (96.87) 2 (3.13)	102 (98.07) 2 (1.93)
**Measures when entering and leaving the house (alone or combined)** Shower Shoe polishing Change of clothes Hand washing Disinfection of clothes	17 (5.43) 51 (16.30) 49 (15.65) 164 (52.39) 32 (10.23)	45 (5.15) 118 (13.52) 174 (19.94) 480 (54.98) 56 (6.41)	4 (3.73) 17 (15.89) 18 (16.83) 62 (57.94) 6 (5.61)	13 (6.67) 31 (15.90) 42 (21.54) 97 (49.74) 12 (6.15)
**Number of contacts plus 15 min and minus 2 m** 0 1–2 3–4 5–6 7–9 10 or more	5 (12.50) 5 (12.50) 15 (37.50) 2 (5.00) 12 (30.00) 1 (2.5)	15 (15.96) 14 (14.90) 46 (48.93) 8 (8.51) 11 (11.70) 0 (0)	4 (18,19) 6 (27.27) 1 (4.54) 11 (50.00) 0 (0) 0 (0)	3 (12.00) 3 (12.00) 6 (24.00) 13 (52.00) 0 (0) 0 (0)
**Type of mask used (alone or combined)** Fabric Hygienic Surgical FFP2 FFP3	59 (19.93) 37 (12.50) 119 (40.20) 77 (26.01) 4 (1.36)	177 (20.12) 99 (11.25) 381 (43.30) 215 (24.43) 8 (0.90)	15 (13.76) 15 (13.76) 45 (41.28) 32 (29.37) 2 (1.83)	20 (10.31) 19 (9.80) 77 (39.69) 72 (37.11) 6 (3.09)
**Virus transmission prevention measures (alone or combined)** Continuous use of mask Avoid crowds No hugs or kisses Frequent hand washing Limit contacts Ozone disinfection	169 (25.80) 130 (19.85) 102 (15.57) 150 (22.90) 98 (14.96) 6 (0.92)	464 (24.46) 383 (20.19) 260 (13.70) 453 (23.89) 325 (17.13) 12 (0.63)	59 (22.27) 49 (18.49) 53 (20.00) 56 (21.13) 47 (17.73) 1 (0.38)	98 (21.03) 93 (19.96) 88 (18.89) 95 (20.38) 87 (18.67) 5 (1.07)

**Table 3:** Multiple logistic regression models of suicidal ideation according to age group and other variables related to confinement.

Variables of the Regression Model for Age Group 1 (≤ 39 Years)						
Variable	β	OF	Wald	*p*	OR	95% CI
Anxiety: Yes	0.691	0.257	7.207	0.007	1.995	1.205–3.304
Loneliness: Yes	−0.340	0.123	7.650	0.006	0.712	0.559–0.906
Stress: Yes	−0.358	0.067	28.851	0.000	0.699	0.613–0.796
Discussions: Yes	−0.238	0.066	13.028	0.000	0.789	0.693–0.897
Drug use: Yes	0.240	0.087	7.622	0.006	1.271	1.072–1.507
Technology dependency: Yes	−0.213	0.029	55.504	0.000	0.808	0.765–0.855
**Variables of the Regression Model for Age Group 2 (** **≥40 years)**						
**Variable**	**β**	**OF**	**Wald**	***p***	**OR**	**95% CI**
Anxiety: Yes	0.691	0.257	7.207	0.007	1.995	1.205–3.304
Loneliness: Yes	−0.340	0.123	7.650	0.006	0.712	0.559–0.906
Stress: Yes	−0.358	0.067	28.851	0.000	0.699	0.613–0.796
Discussions: Yes	−0.238	0.066	13.028	0.000	0.789	0.693–0.897
Drug use: Yes	0.240	0.087	7.622	0.006	1.271	1.072–1.507
Technology dependency: Yes	−0.213	0.029	55.504	0.000	0.808	0.765–0.855

## Data Availability

The data presented in this study are available on request from the corresponding author.
